# Lateral multilayer/monolayer MoS_2_ heterojunction for high performance photodetector applications

**DOI:** 10.1038/s41598-017-04925-w

**Published:** 2017-07-03

**Authors:** Mengxing Sun, Dan Xie, Yilin Sun, Weiwei Li, Changjiu Teng, Jianlong Xu

**Affiliations:** 1 Institute of Microelectronics & Tsinghua National Laboratory for Information Science and Technology (TNList), Tsinghua University, Beijing, 100084 China; 2 Institute of Functional Nano and Soft Materials (FUNSOM), Jiangsu Key Laboratory for Carbon-based Functional Materials and Devices, Soochow University, Suzhou, 215123 Jiangsu Province China

## Abstract

Inspired by the unique, thickness-dependent energy band structure of 2D materials, we study the electronic and optical properties of the photodetector based on the as-exfoliated lateral multilayer/monolayer MoS_2_ heterojunction. Good gate-tunable current-rectifying characteristics are observed with a rectification ratio of 10^3^ at *V*_gs_ = 10 V, which may offer an evidence on the existence of the heterojunction. Upon illumination from ultraviolet to visible light, the multilayer/monolayer MoS_2_ heterojunction shows outstanding photodetective performance, with a photoresponsivity of 10^3^ A/W, a photosensitivity of 1.7 × 10^5^ and a detectivity of 7 × 10^10^ Jones at 470 nm light illumination. Abnormal photoresponse under positive gate voltage is observed and analyzed, which indicates the important role of the heterojunction in the photocurrent generation process. We believe that these results contribute to a better understanding on the fundamental physics of band alignment for multilayer/monolayer MoS_2_ heterojunction and provide us a feasible solution for novel electronic and optoelectronic devices.

## Introduction

Two-dimensional (2D) materials based on atomically thin films of layered semiconductors, such as the family of transition metal dichalcogenides (TMDCs), have exhibited great potentials in various optoelectronic applications^1–5^. Among various TMDCs, MoS_2_ is gaining increasing attention for applications in optoelectronic devices^6–9^, due to the suitable bandgap value, relatively high carrier mobility and high light absorbance^10^. It is interesting that bulk MoS_2_ is semiconducting with an indirect bandgap of 1.2 eV^11^, whereas single-layer MoS_2_ is a direct gap semiconductor with a bandgap of 1.8 eV^12^. In particular, the ability to modulate the band structure by varying the layer numbers allows their unique thickness-dependent electronic and optical properties^2^.

Vertical or lateral semiconductor p-n junctions are the basic building blocks of modern optoelectronic devices^13–15^, such as photodetectors, light emitter diodes and solar cells. Vertical junctions such as WSe_2_/MoS_2_^16^ and black phosphorus/MoS_2_^17^ can be formed by stacking two different 2D materials through Van der Waals forces. However, the band offsets between different TMDCs are pivotal, which could inhibit carrier transport. In addition, impurities are inevitably introduced at the interface during the multiple-transfer process^18^. Within lateral junctions which can be formed via localized chemical doping or electrostatic tuning^3, 19^, the impurities at the interface between p-type and n-type materials can be ignorable. While, multiple complicated fabrication processes are usually required and the band alignment between electrodes and 2D materials is technically challenging. Fortunately, utilizing the band offsets between various numbers of TMDCs layers to form lateral heterojunctions has been proposed in recent years^20, 21^. In 2015, Ali Javey *et al*.^20^ experimentally and theoretically proved the formation of a type-I heterojunction in as-exfoliated MoS_2_ flakes by thickness modulation. Furthermore, Qiaoliang Bao *et al*.^21^ reported a monolayer/bilayer WSe_2_ lateral junction and demonstrated the whole 1L–2 L WSe_2_ junction surface to be active area for photoresponse. However, the photoresponse abilities as well as the photoresponse spectrum of this structure have not been investigated carefully. Also, in such papers, the influence of the junction on photocurrent has not been provided directly.

In this study, electrically tunable as-exfoliated multilayer/monolayer MoS_2_ heterojunction is reported and exhibits good gate-tunable current-rectifying characteristics. Furthermore, we investigate the photoresponse abilities of the heterojunction to different wavelength from ultraviolet (UV) to visible (vis) light. Abnormal photoresponse under positive gate voltage is observed and analyzed, which indicates the important role of the heterojunction in the photocurrent generation process. Upon 470 nm light illumination, the heterojunction shows a photoresponsivity of ~1 × 10^3^ A/W, a photosensitivity of 1.7 × 10^5^ and a detectivity of 7 × 10^10^ Jones which is comparable or higher than most recently reported vertical and lateral heterojunctions^3, 19, 22–25^. This work may provide us a promising heterostructure for novel optoelectronic devices in the future high-performance photodetector applications.

## Results

### Characterization of the multilayer/monolayer MoS_2_ heterojunction

**Figure 1:**
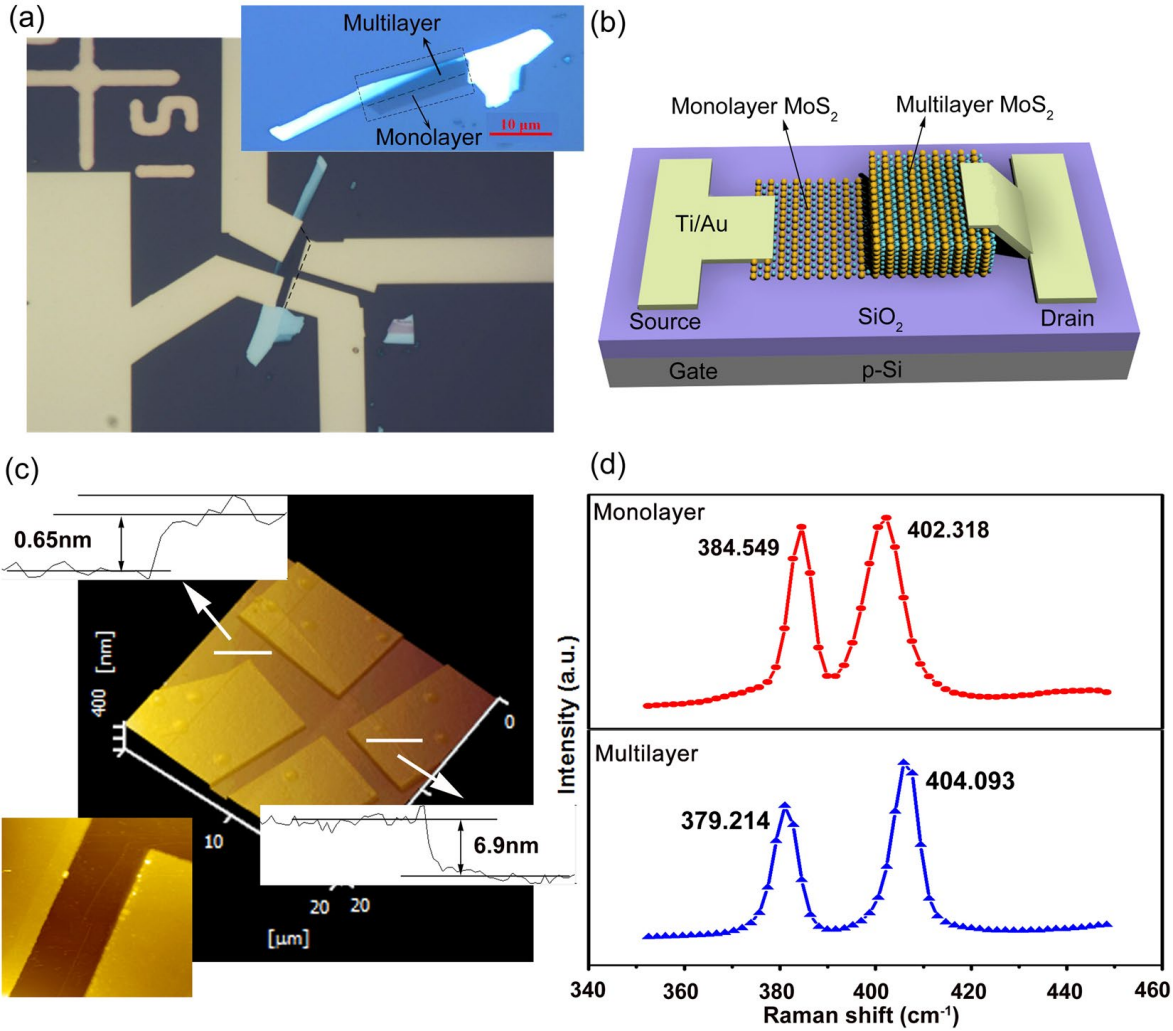
(**a**) The optical microscopy images of MoS_2_ before and after metal deposition. (**b**) The schematic of the photodetector based on multilayer/monolayer MoS_2_ heterojunction. (**c**) The AFM surface morphology of the heterojunctions. (**d**) The Raman spectrum of the monolayer and multilayer MoS_2_.

Figure 1(a) depicts the optical microscopy images of MoS_2_ before and after metal deposition. It can be seen that the colors are different with the layer numbers, which is light gray for monolayer MoS_2_ and dark gray for multilayer MoS_2_. Figure 1(b) shows the schematic of the photodetector based on multilayer/monolayer MoS_2_ heterojunction. In this device, the source electrodes are in contact with the monolayer MoS_2_, the drain electrodes are in contact with the multilayer MoS_2_, and the heavily p-doped Si serves as a global back gate. The thicknesses of the monolayer and multilayer MoS_2_ are ∼0.65 nm and ∼6.9 nm, respectively, as determined from the atomic force microscopy (AFM) measurements shown in Fig. 1(c). From the inset of the Fig. 1(c), an obvious dividing line between monolayer and multilayer MoS_2_ can be observed, which further proves the existence of the heterojunction. The thickness of the MoS_2_ can be also confirmed by the peak positions in Raman spectrum, shown in Fig. 1(d). From the Raman spectrum, we obtain the *E* ^1^_2g_ peak frequencies of 384.549 cm^−1^ (379.214 cm^−1^) and *A*_1g_ peak frequencies of 402.318 cm^−1^ (404.093 cm^−1^) for monolayer (multilayer) MoS_2_ which are consistent with the previous report^26^.

### Electronic properties of the multilayer/monolayer MoS_2_ heterojunction

**Figure 2:**
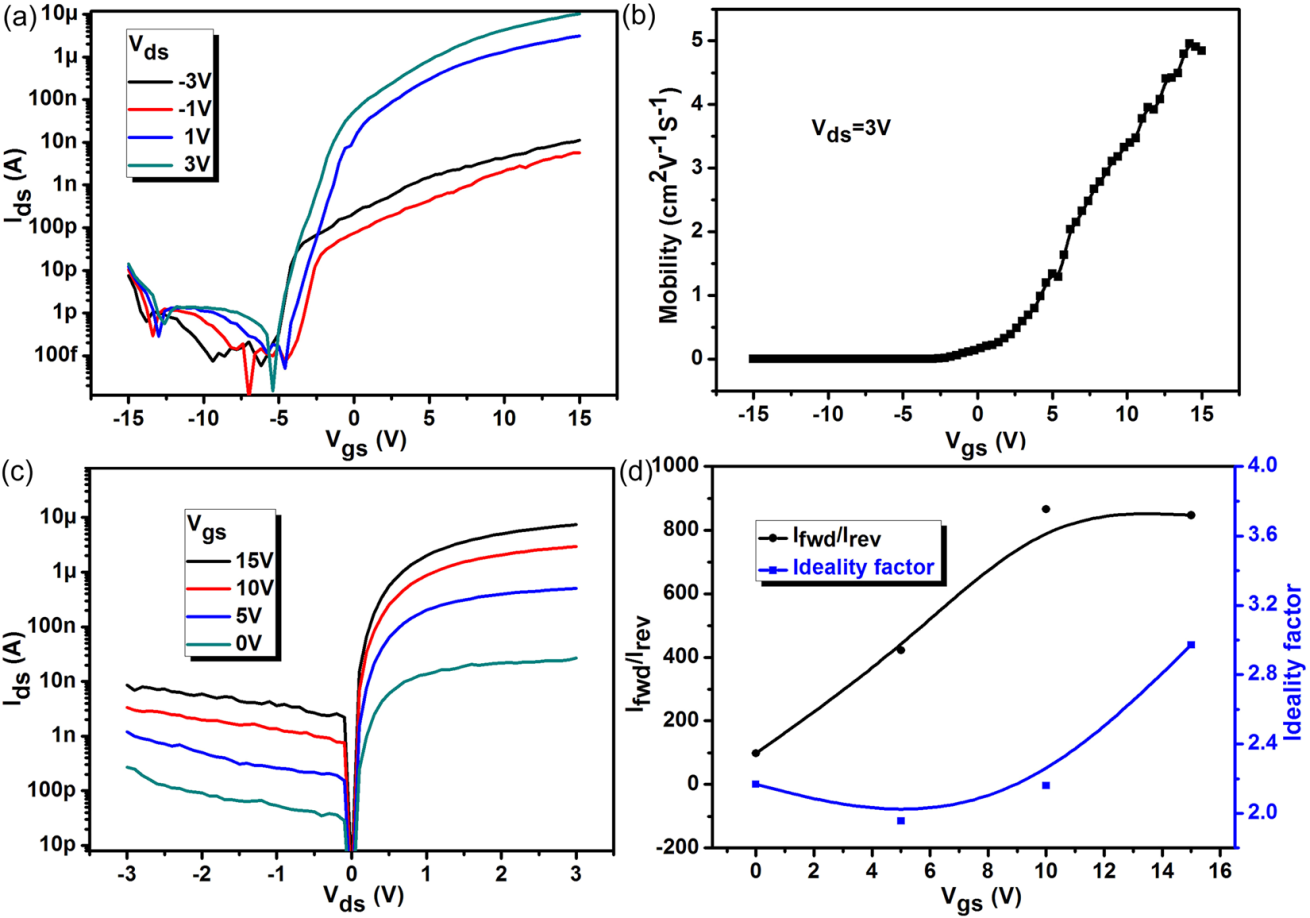
(**a**) Transfer curves of the multilayer/monolayer MoS_2_ heterojunction for both forward and reverse *V*_ds_ bias with back gate modulations. (**b**) Variation of field effect mobility with gate voltage *V*_gs_ obtained from the analysis of experimental transfer characteristics at *V*_ds_ = 3 V. (**c**) Gate tunable *I*_ds_ − *V*_ds_ characteristics of the heterojunction. (**d**) The rectification ratio *I*_fwd_/*I*_rev_ and the ideal factor of the heterojunction as a function of back gate voltage *V*_gs_.

Next, the electrical characteristics of the multilayer/monolayer MoS_2_ heterojunction are studied. Figure 2(a) shows the typical n-type gating characteristics on a semi-log plot with the drain voltage *V*_ds_ changing from −3 V to 3 V. High On-Off current ratio of 10^7^ and a subthreshold swing (*SS* = ∂*V*_gs_/∂ log_10_(*I*_ds_)) close to 300 mV/decade are achieved for this device. The field effect mobility has been extracted from the results in Fig. 2(a) and plotted as a function of *V*_gs_ as shown in Fig. 2(b). The heterojunction shows a typical mobility in the range of 0.1–10 cm^2^ V^−1^ s^−1^, similar to previously reported values for MoS_2_ transistors^27^. Figure 2(c) shows the gate-tunable *I*_ds_ − *V*_ds_ characteristics of the heterojunction on a semi-log plot. It could be concluded that the device exhibits excellent rectifying characteristics and indicates the existence of the multilayer/monolayer MoS_2_ heterojunction. The influence of source/drain Schottky barriers on rectifying behaviors is excluded because of the almost linear output curves of multilayer and monolayer MoS_2_ transistors, as shown in Figure S1 in the supporting information. In Fig. 2(d), the rectification ratio *I*_fwd_/*I*_rev_ (the ratio of the forward/reverse current) of ∼10^3^ is obtained at *V*_ds_ = −3 V/3 V and *V*_gs_ = 10 V. Additionally, the ideal factor of the heterojunction achieves a minimum value of 1.95 with a back gate voltage of 5 V. These strong current-rectifying characteristics and small ideal factor indicate that a high quality of heterojunction has been formed between multilayer and monolayer MoS_2_.

### Photoresponse of the multilayer/monolayer MoS_2_ heterojunction

**Figure 3:**
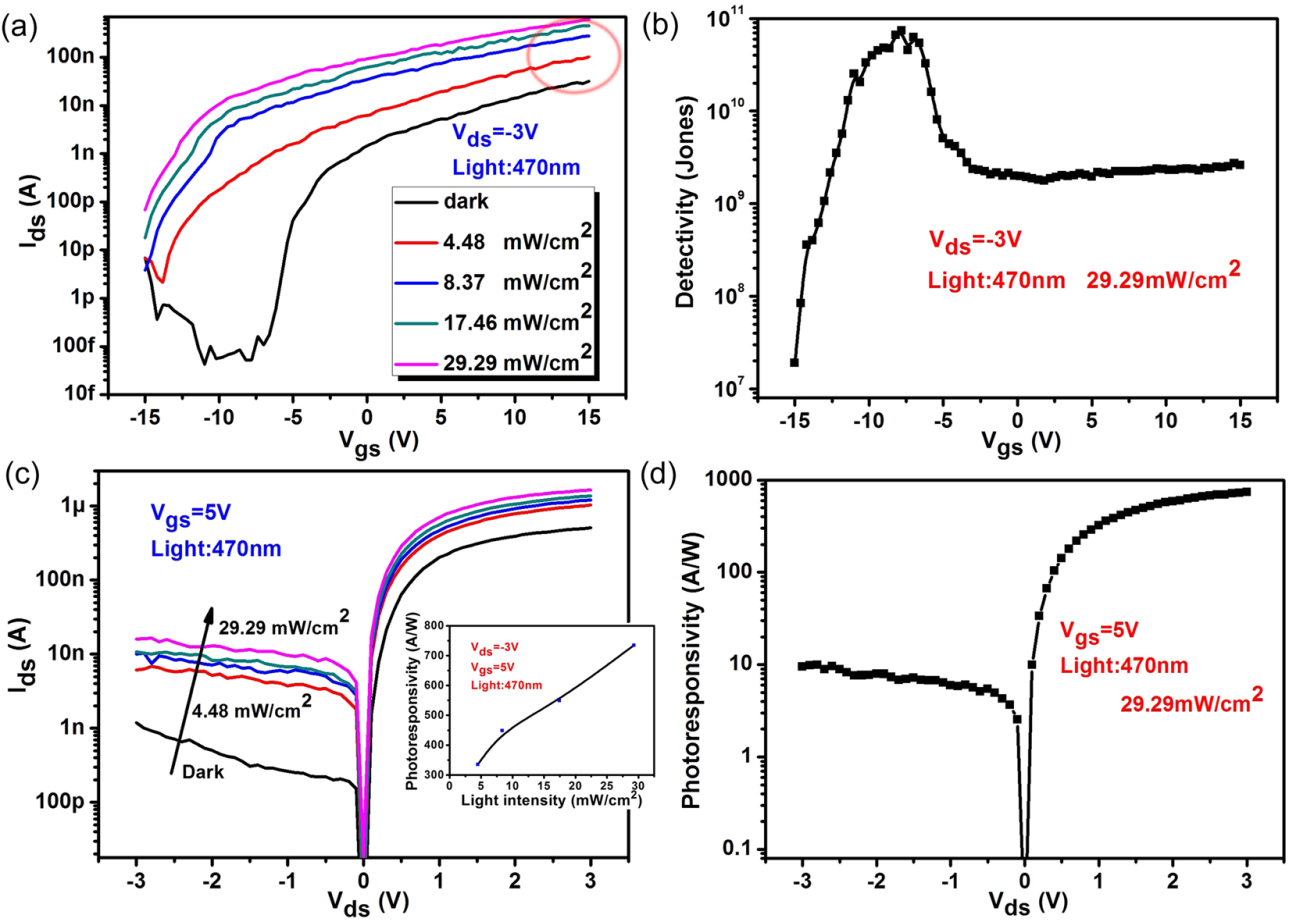
(**a**) *I*_ds_ − *V*_gs_ curves of the multilayer/monolayer MoS_2_ heterojunction with and without 470 nm light illumination. (**b**) The dependence of detectivity on gate voltage. (**c**) *I*_ds_ − *V*_ds_ curves of the multilayer/monolayer MoS_2_ heterojunction with and without 470 nm light illumination. The inset shows the relationship between photoresponsivity and incident power. (**d**) The dependence of photoresponsivity on source-drain voltage.

As high-quality multilayer/monolayer MoS_2_ heterojunction is achieved, the optoelectronic characteristics of the device are then explored. First, we investigate the modulation effects of gate voltage *V*_gs_ on the light detection capabilities. Figure 3(a) shows the transfer curves (*I*_ds_ − *V*_gs_) of the heterojunction under 470 nm light illumination with the light intensity changing from 4.48 mW/cm^2^ to 29.29 mW/cm^2^. The marked increase of current under illumination is observed, indicating the good photoresponse abilities of the device. Furthermore, the n-type characteristic of the heterojunction becomes more pronounced with the increasing of light intensity, which demonstrates the tunable effect of light on electronic behaviors of the heterojunction. To better understand the photoresponse properties of the device, the significant characteristics of the photodetectors for practical applications are concluded, including photosensitivity (*S*, (*I*_light_ − *I*_dark_)/*I*_dark_), photoresponsivity (*R*, (*I*_light_ − *I*_dark_)/*P*_incident_) and detectivity (D*, *A* ^0.5^ *R*/(2q*I*_dark_)^0.5^) where *I*_light_, *I*_dark_, *P*_incident,_ *A* and q is the current under illumination, dark current, incident power, absorbing area and electronic charge, respectively. Figure S2 shows the dependence of *R* and *S* values on gate voltage. Combining the low dark current and high *R*, D* represents the ability of a detector to detect weak optical signals, as show in Fig. 3(b). It can be seen that D* increases and peaks at *V*_gs_ = −7.5 V and then decreases as the gate voltage further increases. The maximum value of D* is about 7 × 10^10^ Jones which is comparable to most reported MoS_2_-based photodetectors^19, 28^. Figure 3(c) displays the output characteristics of the heterojunction under light illumination with different incident powers. The linear dependence of R on incident power can be concluded from the inset of Fig. 3(c). From Fig. 3(d), the value of R increases as *V*_ds_ increases and reaches the maximum value of *R* is about 10^3^ A/W at *V*_ds_ = 3 V, which is comparable or higher than most recently reported vertical and lateral heterojunctions^3, 19, 22–25^.

**Figure 4:**
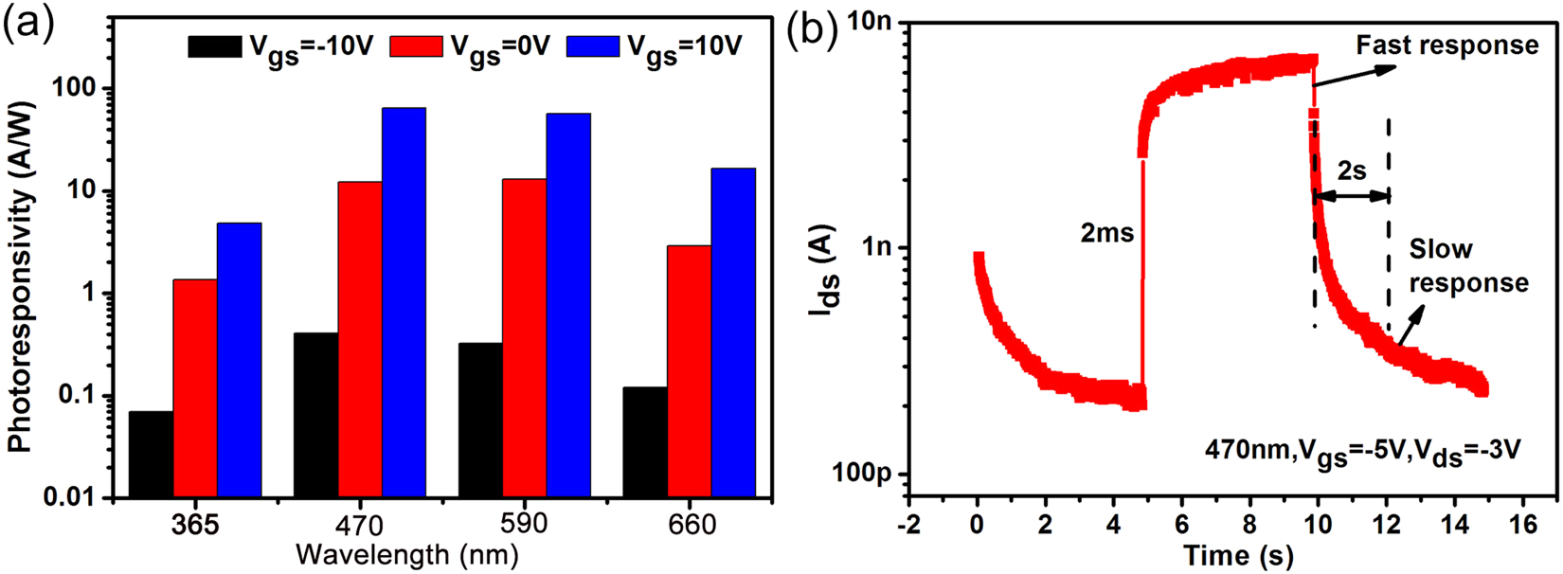
(**a**) The photoresponsivity under different light wavelengths (typically for 365 nm, 470 nm, 590 nm, 660 nm) with *V*_gs_ changing from −10 V to +10 V. (**b**) Time-resolved photoresponse of the heterojunction under 470 nm light illumination, recorded for *V*_gs_ = −5 V, *V*_ds_ = −3 V.

To apply the multilayer/monolayer MoS_2_ heterojunction to a broadband photodetector^29, 30^, the photoresponse of the device on other light wavelength has also been investigated. The different photoresponsivities of the device on various wavelength (typically for 365 nm, 470 nm, 590 nm, 660 nm) are shown in Fig. 4(a). The device exhibits a broadband photoresponse from ultraviolet to visible light and shows a slightly larger *R* under 470 nm light illumination. However, due to the relatively weak infrared light absorption^22^, there is no obvious photoresponse on infrared region. An interdigitated finger structure and laser light source are suggested for more accurate investigation on infrared region. Response speed is also one of the key figure of merits for a photodetector, particularly for that utilized in optical communication, imaging, and so on. Figure 4(b) shows the time-resolved measurement to study its photoresponse dynamics. The response is characterized by a typical rise time *τ*_rise_ of 2 ms and decay time *τ*_decay_ of 2 s. The fast rise time is induced by the depletion region of the heterojunction and Schottky barriers of the source/drain contact. However, due to the existence of adsorbates, defects or charge impurities in surrounded MoS_2_ materials, a slow relaxation speed might be observed in the decay time. To reduce the response time, a more independent environment of channel is needed. Additionally, good photostability over multiple cycles of the device can be concluded from Figure S3.

### Working mechanism of the multilayer/monolayer MoS_2_ heterojunction

**Figure 5:**
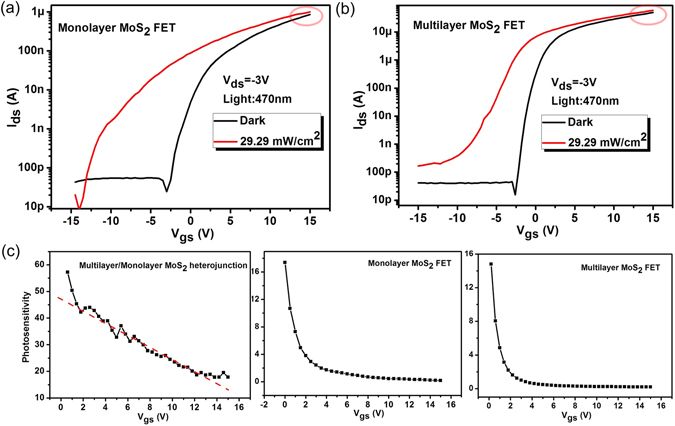
(**a** and **b**) The transfer curves of monolayer MoS_2_ and multilayer MoS_2_ photo-transistors. (**c**) The photosensitivity changes of three kinds of devices with the change of gate voltage.

To analyze the heterojunction effect on the photoresponse behaviors, the transfer curves of monolayer MoS_2_ and multilayer MoS_2_ photo-transistors are plotted in Fig. 5(a) and (b) respectively. Ignorable photoresponses are observed in the on state (*V*_gs_ = 15 V) of the devices. The similar phenomenon has also been reported in the literatures^2, 22^. However, obvious photoresponse behaviors in the heterojunction are observed at the forward gate bias voltage (Fig. 3(a)). Furthermore, as shown in Fig. 5(c), the *S* of the heterojunction shows a linear dependence on gate voltage. Differently, the *S* of multilayer MoS_2_ transistor and monolayer MoS_2_ transistor both decrease exponentially as the gate voltage increases. The different photoresponse characteristics of two kinds of devices might be owing to the existence of the heterojunction and the reason will be discussed in the next part.

**Figure 6:**
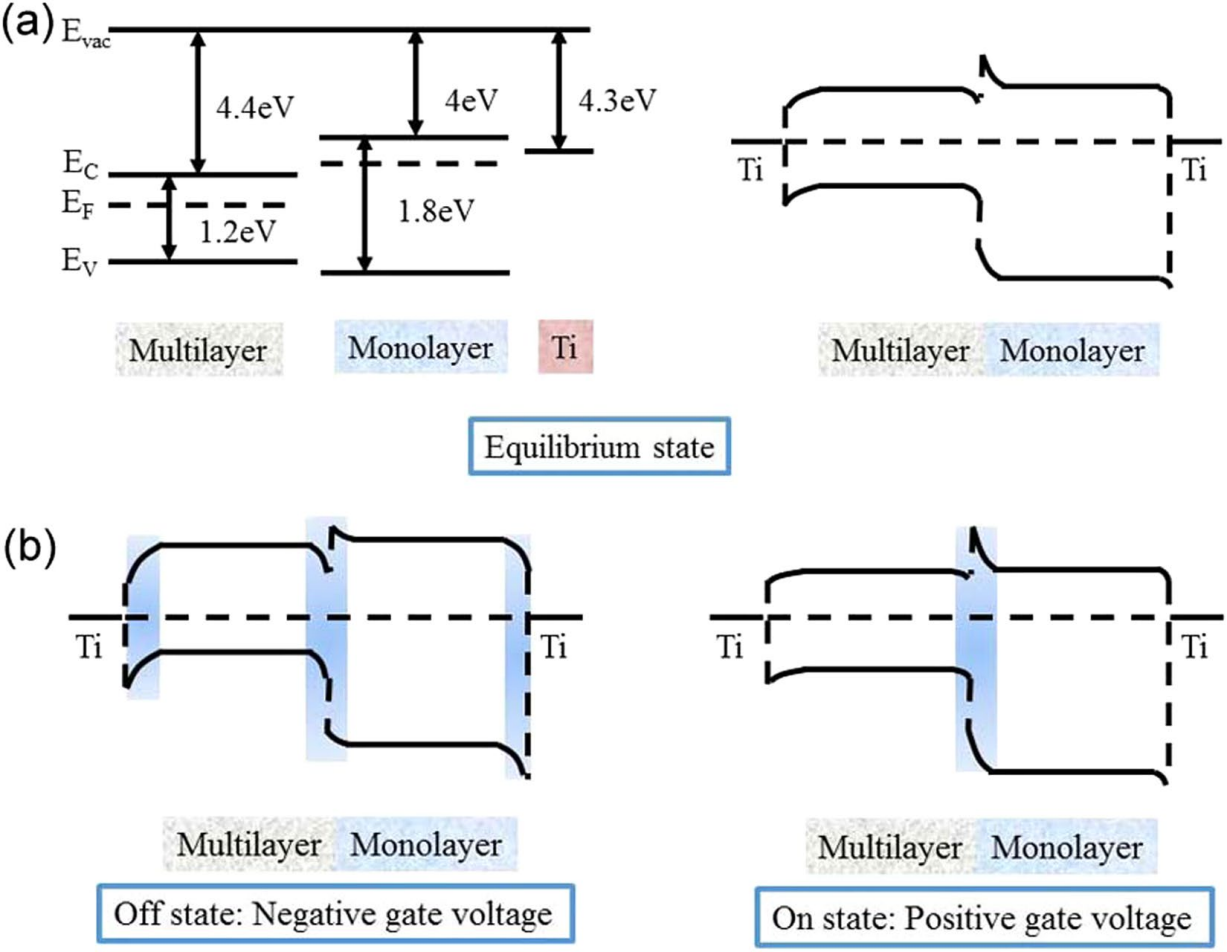
(**a**) The band diagram of the heterojunction in equilibrium state. (**b**) The band diagram of the heterojunction in off state (negative gate voltage) and on state (positive gate voltage) with the *V*_ds_ = 0 V. The blue regions represent the effective photosensitive areas.

To better understand the working mechanism of the heterojunction, the energy band diagram is shown in Fig. 6. According to the reported experimental and theoretical bandgap values for monolayer and multilayer MoS_2_^20, 31^, a type-I heterojunction in equilibrium state is expected as depicted in the qualitative band diagram of Fig. 6(a). Simultaneously, Schottky barriers between MoS_2_ and source/drain metal are formed^32^. Fig. 6(b) exhibits the typical band alignments in off state (negative gate voltage) and on state (positive gate voltage). Under negative gate voltage, the conduction band (E_C_) and valance band (E_V_) are pulled downward, which induces MoS_2_/Ti Schottky barriers. At this condition, the effective photosensitive areas (blue regions in Fig. 6(b)) consist of MoS_2_/Ti Schottky barriers and multilayer/monolayer MoS_2_ heterojunction. As the gate voltage moves toward positive values, MoS_2_/Ti Schottky contact changes to the Ohmic contact. Correspondingly, photovoltage effect which is induced by the contact barriers will be weakened. However, multilayer/monolayer MoS_2_ heterojunction still plays an important role in photoresponse process. So these devices exhibit different decreasing trend in Fig. 5c. As a conclusion, the good photoresponse of the multilayer/monolayer MoS_2_ heterojunction might be derived not only from the effect of the Schottky barrier in the MoS_2_/metal contact but also from the effect of the build-in field in the heterojunction.

## Discussion

The lateral multilayer/monolayer MoS_2_ heterojunction is fabricated and the electronic and optical characteristics are investigated under the gate modulation. The lateral 2D heterojunction possesses a high On-Off current ratio of 10^7^ and good current-rectifying characteristics with a high rectification ratio of 10^3^ and a small ideality factor of 1.95 in the dark, revealing the high quality of the heterojunction. As a photodetector, the multilayer/monolayer MoS_2_ heterojunction exhibits good photodetection capabilities upon the illumination from ultraviolet to visible light. Under 470 nm light illumination, the device shows a maximum photoresponsivity of 10^3^ A/W, a high photosensivity of 10^5^ and detectivity of 7 × 10^10^ Jones. This work could offer an interesting platform for fundamental investigations of lateral multilayer/monolayer TMDCs heterojunctions, and will be valuable for fabricating flexible and transparent optoelectronic devices in the future.

## Method

A multilayer/monolayer MoS_2_ flake was obtained from a bulk crystal by mechanical exfoliation method and transferred to a highly p-doped Si (100) substrates with 90 nm thermal oxide as shown in the inset of Fig. 1(a). Metal source/drain (S/D) contacts are subsequently formed with source contact on the monolayer region and the other on the multilayer region of the MoS_2_ flake. Then, electron-beam lithography (EBL) was used to pattern the source/drain contacts, followed by thermal evaporation of Ti/Au (10/50 nm) electrodes and lift-off process. The resulting structure is shown in Fig. 1(a) with channel length *L* of 3 μm and width *W* of 7.4 μm. Atomic force microscope (AFM, SPA 500, Seiko Instruments Inc.) and Raman spectroscopy (RM-1000, Renishaw) with a wavelength of 532 nm were used to confirm the layer number of MoS_2_ flakes. The electronic and optical properties of multilayer/monolayer MoS_2_ heterojunction were characterized with an Agilent B1500 parameter analyzer at room temperature in air ambient. The monochromic lights with different wavelengths were provided by CEL-LEDS35 LED illuminant system (CEAULIGHT).

## Electronic supplementary material

Supplementary Information
