# Supplementary material for: Lateral multilayer/monolayer MoS2 heterojunction for high performance photodetector applications
Source: Sci Rep. 2017 Jul 3;7:4505. doi: 10.1038/s41598-017-04925-w (PMC5495793; doi:10.1038/s41598-017-04925-w)
Supplement: Supplementary file 1 — Supplementary Information [file 41598_2017_4925_MOESM1_ESM.doc]

**Supplementary Information**

Lateral multilayer/monolayer MoS_2_ heterojunction for high performance photodetector applications

*Mengxing Sun^1^, Dan Xie ^1^*, Yilin Sun^1^, Weiwei Li^1^, Changjiu Teng^1^ and Jianlong Xu^2^*


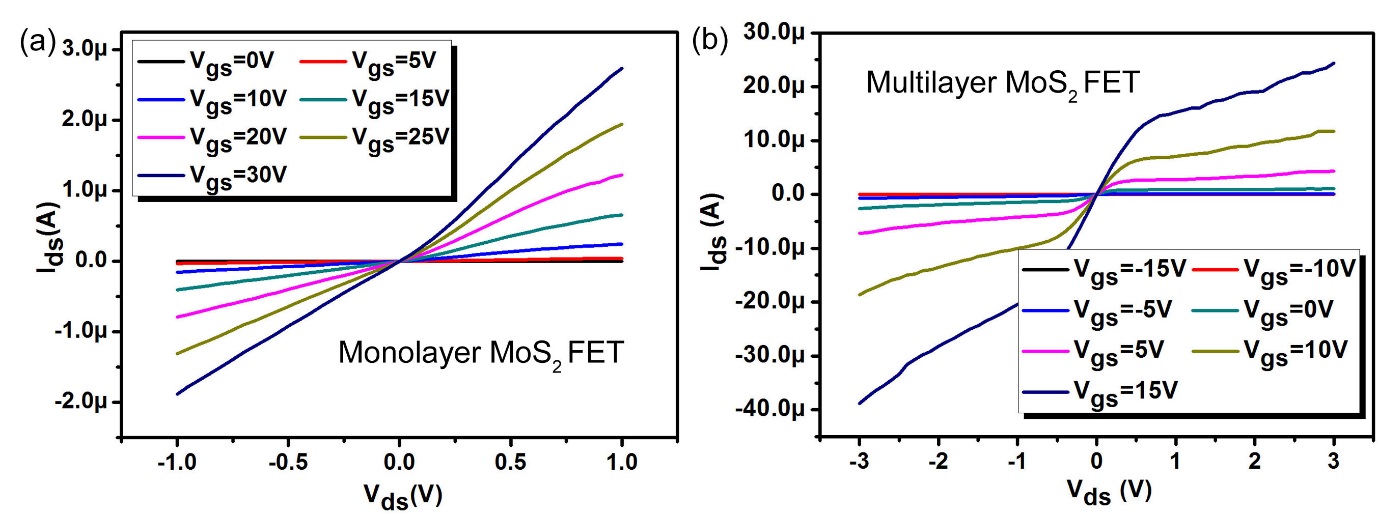


**Figure S1**. (a) The output curves of monolayer MoS_2_ FET under the gate modulation. (b) The output curves of multilayer MoS_2_ FET under the gate modulation.


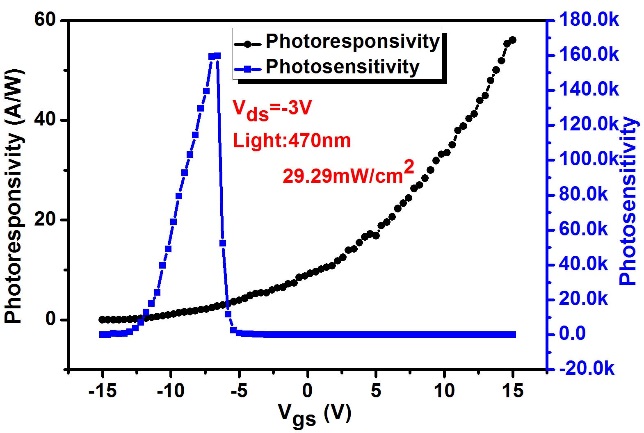


**Figure S2**. The dependence of *S* and *R* on the gate voltage of the heterojunction.


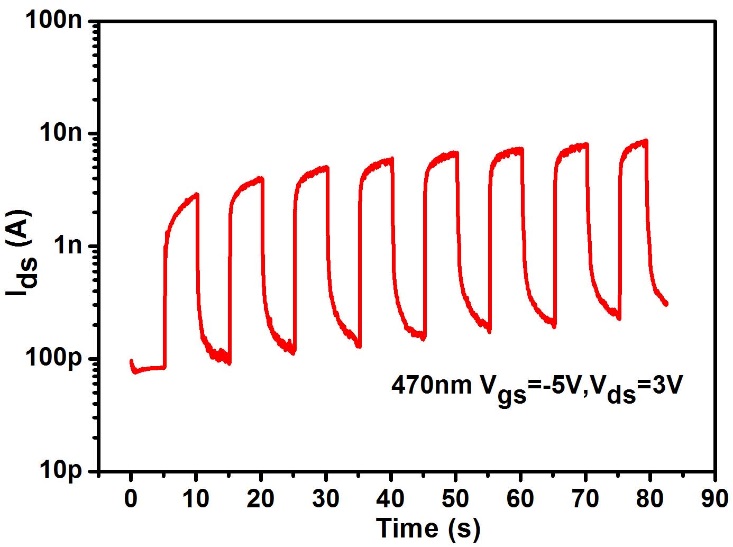


**Figure S3.** Current-time cycles of the multilayer/monolayer MoS_2_ heterojunction.
